# Analyses of the relationship between hyperuricemia and osteoporosis

**DOI:** 10.1038/s41598-021-91570-z

**Published:** 2021-06-08

**Authors:** Jung Woo Lee, Bong Cheol Kwon, Hyo Geun Choi

**Affiliations:** 1 Department of Orthopaedic Surgery, Yonsei University Wonju College of Medicine, Wonju, Korea; 2 Department of Orthopaedic Surgery, Hallym University College of Medicine, Anyang, Korea; 3 Department of Otorhinolaryngology-Head and Neck Surgery, Hallym University Sacred Heart Hospital, Hallym University College of Medicine, 22, Gwanpyeong-ro 170beon-gil, Dongan-gu, Anyang-si, 14068 Gyeonggi-do Korea; 4 Hallym Data Science Laboratory, Hallym University College of Medicine, Anyang, Korea

**Keywords:** Endocrinology, Medical research, Risk factors

## Abstract

The aim of the present study was to evaluate the association between hyperuricemia and osteoporosis in a Korean population. Data from participants of the Korean Genome and Epidemiology Study who were ≥ 40 years old were collected from 2004 to 2016. Among 173,209 participants, 11,781 with hyperuricemia (> 7.0 mg/dL in men and > 6.0 mg/dL in women) and 156,580 controls were selected based on serum measurements. Odds ratios (ORs) of osteoporosis between individuals with hyperuricemia and controls were analyzed using a logistic regression model. In the adjusted model, age, sex, income group, body mass index, smoking, alcohol consumption, hypertension, diabetes mellitus, hyperlipidemia history and nutritional intake were adjusted. The adjusted OR (aOR) of osteoporosis was 0.79 [95% confidence interval (CI) = 0.71–0.87, *P* < 0.001]. In subgroup analyses according to age and sex, statistical significance was observed in men > 60 years old and in women > 50 years old. In another subgroup analysis according to past medical history, significant differences were found according to hypertension (aOR = 0.83, 95% CI = 0.73–0.94, and 0.75, 95% CI = 0.64–0.87), diabetes mellitus (aOR = 0.77, 95% CI = 0.69–0.86), and hyperlipidemia (aOR = 0.74, 95% CI = 0.61–0.89, and 0.81, 95% CI = 0.72–0.91). This study demonstrated that hyperuricemia was associated with a decreased risk of osteoporosis.

## Introduction

Hyperuricemia refers to elevated uric acid in the blood. Hyperuricemia that is caused by the overproduction of urate or, more commonly, by renal urate underexcretion is necessary but not sufficient to cause gout^1^. The prevalence of hyperuricemia has increased slightly, from 19.1% between 1988 and 1994 to 21.5% between 2007 and 2008 in the National Health and Nutrition Examination Survey (NHANES)^2^. Approximately 47.1 million adults in the US met the sex-specific criteria for hyperuricemia from 2015 to 2016^3^. With increasing levels of hyperuricemia, there were graded increases in the prevalence of comorbidities^4^.

Osteoporosis is a metabolic bone disorder characterized by low bone mineral density (BMD) and increased skeletal fragility. Osteoporosis is common in the elderly population, and the prevalence of osteoporosis in individuals aged ≥ 50 years is estimated to be 16.0% in men and 29.9% in women in the United States^5^. Secondary causes of osteoporosis include chronic treatment with glucocorticoids, gastrointestinal disorders, diabetes mellitus, rheumatoid arthritis, liver disease, gluten enteropathy, multiple myeloma and other hematologic disorders^6^.

There has been an increase in the evidence supporting the favorable and protective effect of higher uric acid on bone metabolism in the past decade^7–18^. Recent studies revealed a positive relationship between uric acid and lumbar BMD among most adolescents and old adults^19,20^. Meta-analysis showed that subjects with hyperuricemia had significantly higher BMD values in the spine [standardized mean differences (SMD) 0.29, 95% confidence intervals (CI) 0.22–0.35], total hip (SMD 0.29, 95% CI 0.24–0.34) and femoral neck (SMD 0.25, 95% CI 0.74–0.92)^21^. However, Zhang et al. found that serum uric acid was independent of BMD after adjusting for confounding variables and applying multivariate analysis in a sample of 6579 American adult individuals^22^. Furthermore, Mendelian randomization analysis did not support a causal association between uric acid level and total femur (β = − 0.29), femoral neck (β = − 0.27), and spine (β = 0.08) BMD in people from the United States^23^; lumbar spine (β = 0.385), hip (β = 0.191), or femoral neck (β = 0.194) BMD in Chinese people^24^; or lumbar spine (β = − 0.700) BMD in Korean people^25^.

Although previous studies support a protective role for uric acid in bone metabolism disorders, the majority of the studies were conducted in a single institution^10,26^, restricted to a single sex^14^, limited to old participants^8,9^ or the sample sizes were small^7,11^. No study stratified the groups by sex and age, and no study performed subgroup analyses according to past medical histories. The aim of this study was to evaluate the association between hyperuricemia and osteoporosis in a nationwide population-based cross-sectional study with a significantly larger population than before. Blood tests were also performed on all asymptomatic people, and hyperuricemia was also found among those who would not have visited the hospital. In this study, we estimated the odds ratios (ORs) of osteoporosis in hyperuricemia patients compared to controls.

## Materials and methods

### Study population and data collection

The Institutional Review Board and Ethics Committee of Hallym University Sacred Heart Hospital (IRB No. 2019-02-020) approved the use of these data. The requirement for written informed consent was waived by the Institutional Review Board and Ethics Committee of Hallym University Sacred Heart Hospital. All methods were performed in accordance with the relevant guidelines and regulations. This prospective cross-sectional study relied on data from the Korean Genome and Epidemiology Study (KoGES) from 2004 through 2016. A detailed description of these data was provided in a previous study^27^. Among the KoGES Consortium, we used KoGES health examinee (HEXA) data consisting of participants living in urban areas who were ≥ 40 years old. The data consisted of the baseline data from 2004 to 2013 and follow-up data from 2012 to 2016.

### Participant selection

**Figure 1:**
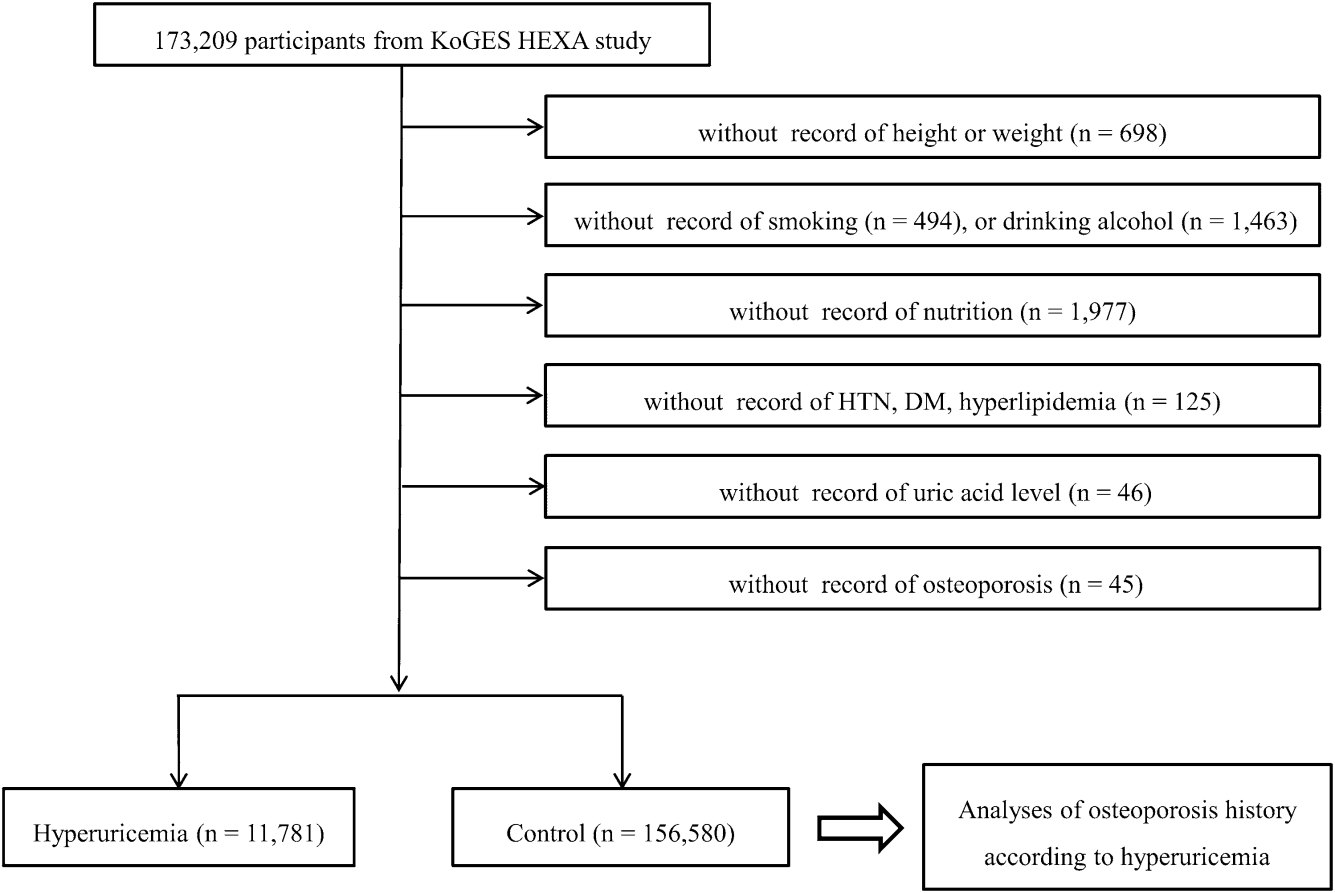
A schematic illustration of the participant selection process used in the present study. Of a total of 173,209 participants, 11,781 hyperuricemia patients and 156,580 control participants were included.

Among 173,209 participants, we excluded participants who lacked records of height or weight (n = 698), smoking history (n = 494), alcohol consumption habits (n = 1,463), nutrition (n = 1,977), hypertension, diabetes mellitus, and hyperlipidemia history (n = 125), uric acid measurements (n = 46), and osteoporosis history (n = 45). Finally, 11,781 hyperuricemia and 156,580 control (nonhyperuricemia) participants were selected (Fig. 1). Then, we analyzed the histories of osteoporosis among the hyperuricemia and control participants.

### Survey

The participants were asked about their previous histories of hypertension, diabetes mellitus, hyperlipidemia, and osteoporosis by trained interviewers. We defined hyperuricemia as uric acid > 7.0 mg/dL in men^2^ and > 6.0 mg/dL in women^28^ following previous studies. Body mass index (BMI) was calculated as kg/m^2^ using the health checkup data. Smoking history was categorized as nonsmokers (< 100 cigarettes over their entire life), past smokers (quit more than one year ago), and current smokers. Participants were categorized according to their alcohol consumption habits as nondrinkers, past drinkers, and current drinkers. Their nutritional intake [total calories (kcal/day), protein (g/day), fat (g/day), carbohydrates (g/day), calcium (mg/d), and phosphorus (mg/d)] was surveyed by a food-frequency questionnaire that was validated by a previous study^29^. The income group was categorized as nonrespondent, low income (< ~ $2000 per month), middle income (~ $2000–$3999 per month), and high income (~ ≥ $4000 per month) by their household income.

### Statistical analyses

Chi-square tests were used to compare the rates of sex, income group, smoking, alcohol consumption, and hypertension, diabetes mellitus, and hyperlipidemia history^30^. Independent t-tests were used to compare age, BMI, and nutritional intake^7^.

To analyze the OR of hyperuricemia for osteoporosis, a logistic regression model was used^13^. Crude and adjusted models [age, sex, income group, BMI, smoking, alcohol consumption, hypertension, diabetes mellitus, and hyperlipidemia history and nutritional intake (total calorie, protein, fat, carbohydrate, calcium, and phosphorous intake)] were used^31^. In the subgroup analyses according to age and the sex, we divided age into 10-year increments.

Two-tailed analyses were conducted, and P values less than 0.05 were considered to indicate significance^30^. The results were statistically analyzed using SPSS v. 24.0 (IBM, Armonk, NY, USA).

## Results

The general characteristics of participants were different between hyperuricemia and control participants (Table 1).

**Table 1:** General characteristics of participants.

Characteristics	Total participants		*P* value
	Hyperuricemia	Control	
Age (mean, SD, y)	54.9 (8.7)	53.0 (8.3)	< 0.001*
Sex (n, %)			< 0.001*
Men	7775 (66.0)	50,002 (31.9)	
Women	4006 (34.0)	106,578 (68.1)	
BMI (mean, SD, kg/m^2^)	25.5 (3.0)	23.8 (2.9)	< 0.001*
Income (n, %)			< 0.001*
Missing, no response	2086 (17.7)	24,549 (15.7)	
Lowest	3240 (27.5)	42,806 (27.3)	
Middle	4018 (34.1)	56,589 (36.1)	
Highest	2437 (20.7)	32,636 (20.8)	
Smoking status (n, %)			< 0.001*
Nonsmoker	5774 (49.0)	116,888 (74.7)	
Past smoker	3507 (29.8)	21,218 (13.6)	
Current smoker	2500 (21.2)	18,474 (11.8)	
Alcohol consumption (n, %)			< 0.001*
Non drinker	4,000 (34.0)	81,519 (52.1)	
Past drinker	734 (6.2)	5,899 (3.8)	
Current drinker	7047 (59.8)	69,162 (44.2)	
Hypertension	4771 (40.5)	32,706 (20.9)	< 0.001*
Diabetes mellitus	1240 (10.5)	12,049 (7.7)	< 0.001*
Hyperlipidemia	2037 (17.3)	20,156 (12.9)	< 0.001*
Nutritional intake			
Total calories (kcal/d)	1786.6 (595.9)	1756.7 (590.3)	< 0.001*
Protein (g/d)	61.1 (27.6)	59.8 (27.2)	< 0.001*
Fat (g/d)	29.0 (19.0)	28.1 (18.7)	< 0.001*
Carbohydrate (g/d)	315.5 (96.6)	312.0 (96.5)	< 0.001*
Calcium (mg/d)	431.9 (262.3)	453.5 (276.9)	< 0.001*
Phosphorus (mg/d)	902.4 (375.6)	900.5 (379.5)	0.600
Osteoporosis (n, %)	502 (4.3)	11,020 (7.0)	< 0.001*

*Independent T-test or Chi-square test. Significance at *P* < 0.05.

The adjusted OR (aOR) of hyperuricemia for osteoporosis was 0.79 [95% confidence interval (CI) = 0.71–0.87, *P* < 0.001, Table 2]. In subgroup analyses, the finding was consistent only in older women. The aORs were 0.86 (95% CI = 0.41–1.82) in ≤ 50-year-old men, 1.18 (95% CI = 0.80–1.72) in ≤ 50-year-old women, 1.08 (95% CI = 0.68–1.71) in 51–60 years old men, 0.76 women (95% CI = 0.64–0.90, *P* = 0.002) in 51–60 years old, 0.60 (95% CI = 0.38–0.94, *P* = 0.026) in > 60-year-old men, and 0.79 (95% CI = 0.69–0.91, *P* = 0.001) in > 60-year-old women. Statistical significance was observed in men > 60 years old and in women > 50 years old.

**Table 2:** Crude and adjusted odd ratios (95% confidence interval) of hyperuricemia for osteoporosis.

Characteristics	Odd ratios for osteoporosis			
	Crude	*P* value	Adjusted^†^	*P* value
**Total participants (n = 168,361)**				
Hyperuricemia	0.59 (0.54–0.64)	< 0.001*	0.79 (0.71–0.87)	< 0.001*
Control	1.00		1.00	
**Age ≤ 52 years old, men (n = 26,485)**				
Hyperuricemia	0.62 (0.30–1.29)	0.203	0.67 (0.32–1.39)	0.282
Control	1.00		1.00	
**Age ≤ 52 years old, women (n = 57,795)**				
Hyperuricemia	1.14 (0.85–1.53)	0.370	1.01 (0.75–1.37)	0.935
Control	1.00		1.00	
**Age ≥ 53 years old, men (n = 31,292)**				
Hyperuricemia	0.77 (0.56–1.06)	0.104	0.81 (0.59–1.12)	0.198
Control	1.00		1.00	
**Age ≥ 53 years old, women (n = 52,789)**				
Hyperuricemia	0.80 (0.72–0.90)	< 0.001*	0.77 (0.69–0.86)	< 0.001*
Control	1.00		1.00	

*Logistic regression model, Significance at *P* < 0.05.

^†^Models adjusted for age, sex, income group, BMI, smoking, alcohol consumption, hypertension, diabetes mellitus, hyperlipidemia histories and nutritional intake (total calories, protein, fat, carbohydrate intake, calcium, and phosphorous intake).

In another subgroup analysis according to the past medical histories of hypertension, diabetes mellitus, and hyperlipidemia, the results showed statistical significance except for in the participant with diabetes mellitus (Table 3).

**Table 3:** Crude and adjusted odd ratios (95% confidence interval) of hyperuricemia for osteoporosis according their past medical histories.

Characteristics	Odd ratios for osteoporosis			
	Crude	*P* value	Adjusted^†^	*P* value
**With hypertension (n = 37,477)**				
Hyperuricemia	0.59 (0.53–0.67)	< 0.001*	0.83 (0.73–0.94)	0.005*
Control	1.00		1.00	
**Without hypertension (n = 130,884)**				
Hyperuricemia	0.45 (0.39–0.51)	< 0.001*	0.75 (0.64–0.87)	< 0.001*
Control	1.00		1.00	
**With diabetes mellitus (n = 13,289)**				
Hyperuricemia	0.83 (0.66–1.04)	0.106	0.88 (0.69–1.13)	0.315
Control	1.00		1.00	
**Without diabetes mellitus (n = 155,072)**				
Hyperuricemia	0.55 (0.50–0.61)	< 0.001*	0.77 (0.69–0.86)	< 0.001*
Control	1.00		1.00	
**With hyperlipidemia (n = 22,193)**				
Hyperuricemia	0.48 (0.41–0.57)	< 0.001*	0.74 (0.61–0.89)	0.002*
Control	1.00		1.00	
**Without hyperlipidemia (n = 146,168)**				
Hyperuricemia	0.59 (0.53–0.66)	< 0.001*	0.81 (0.72–0.91)	< 0.001*
Control	1.00		1.00	

*Logistic regression model, Significance at *P* < 0.05.

^†^Models adjusted for age, sex, income group, BMI, smoking, alcohol consumption, hypertension, diabetes mellitus, hyperlipidemia histories and nutritional intake (total calories, protein, fat, carbohydrate intake, calcium, and phosphorous intake).

## Discussion

In our study, the OR for osteoporosis in the hyperuricemia group was lower than that in the control group (aOR 0.79, 95% CI = 0.71–0.87). The strengths of this study are that it is the largest study investigating risk factors in not only subjects with hyperuricemia but also controls with normal serum uric acid levels. This cross-sectional study included asymptomatic participants regardless of their past medical history, possibly revealing undiagnosed hyperuricemia patients. Additionally, the inclusion of a sufficient number of participants allowed us to define hyperuricemia with > 7.0 mg/dL in men and > 6.0 mg/dL in women, which are clinically meaningful reference values.

In our study, the prevalence of hyperuricemia was 7.5% (13.5% in men and 3.8% in women), which was slightly lower than the 11.4% (17.0% in men and 5.9% in women) identified in a sample of 5,548 individuals from the general Korean population^32^ and much lower than that of the general American population (21.4% from 2007 to 2008)^2^. Our study included participants ≥ 40 years old, and a previous report included participants ≥ 19 years old^32^. As the prevalence was high in individuals under 40 years old in both sexes in a previous report^32^, age was probably a major contributor to the differences in prevalence rates.

Since elderly people have the coexistence of common conditions of hypertension, diabetes mellitus and hyperlipidemia, these conditions may confound the association between hyperuricemia and osteoporosis. To our knowledge, this is the first study that has analyzed subgroups in association with hyperuricemia and osteoporosis. Many studies have reported that smoking^30^, alcohol consumption^33^ and nutritional intake^34^ are factors influencing bone metabolism. In our study, there were still significant findings after adjusting for confounders.

To our knowledge, our study had the largest sample with 11,781 hyperuricemia and 156,580 control participants, followed by 607 hyperuricemia and 4,592 control participants in the study by Yan et al.^13^. Most previous studies analyzed the association between serum uric acid levels and BMD rather than evaluating the association between hyperuricemia and osteoporosis. In recent studies, the categories of serum uric acid were not described^7,26,35^, and the authors divided participants according to their median serum uric acid level^11^, tertiles^8^, quantiles^15,20^ or quintiles^10^. Few studies have included subgroups with serum uric acid levels > 7 mg/dL, indicating hyperuricemia^9,10^, but the subgroup was as small as 25/1080^9^ and the participants were limited to men aged 40 to 60 years^10^.

The aOR of 0.79 was lower than that for females reported in a preceding study conducted with a Chinese cohort (aOR 0.874, 95% CI = 0.824–0.928)^13^. We believe that these differences may be due to the study design and included participants. We excluded participants who were younger than 40 years at the time of the diagnosis of hyperuricemia and divided age into 10-year increments. However, a previous Chinese study included participants of all ages and did not divide groups by age^13^. In our study, we used an adjusted model that adjusted not only for age but also for sex, income group, BMI, smoking, alcohol consumption, hypertension, diabetes mellitus, hyperlipidemia and nutritional intake. Four more studies presented results with ORs^15–18^ but used the median or quartiles as subgroups rather than the hyperuricemia cutoff. In another matched cohort study (with 36,458 with gout and 71,602 without gout), the gout cohort showed a 1.2-fold increase in the incidence of osteoporosis (adjusted hazard ratio 1.2, 95% CI = 1.06–1.35)^36^. However, since the study examined the medical claim of gout, the exact level of uric acid is unknown.

According to past medical history, hyperuricemia participants with diabetes mellitus did not show a statistically significant decrease in OR in the present study. Type 2 diabetes mellitus affects bone metabolism and strength in a direct way, and certain antidiabetic medications affect bone metabolism^37^. Hyperuricemia can aggravate the progression of diabetes and lead to bone fragility; thus, hyperuricemia can indirectly accelerate bone loss in type 2 diabetes patients^38^. In a study with Chinese type 2 diabetes mellitus patients, the association of uric acid with osteoporosis varied by sex, BMI and skeletal site^39^. In their study, multiple linear regression analysis demonstrated a significant association between serum uric acid levels and BMD in the BMI < 25 group (beta: 0.146 to 0.218). Further investigations are needed to explain this issue.

In the subgroup analysis in our study, the OR of hyperuricemia for osteoporosis was significantly lower than that of the control group in older men (> 60 years) and women (> 50 years). Statistically significant findings were found for men and women of different age groups. The prevalence of hyperuricemia and osteoporosis differs by age and sex distribution; the prevalence of hyperuricemia is higher in men than in women^32^, and osteoporosis is more prevalent in older women^5^. This association indicates that hyperuricemia may have a protective effect against osteoporosis in older women. Uric acid levels increase more significantly in postmenopausal females than in males^40^. It was hypothesized that the antioxidant effect of uric acid antagonizes oxidative stress-induced bone metabolism^12^. Although a positive association between uric acid and BMD was observed in some studies with males^10,18^, the association between hyperuricemia and osteoporosis was not studied in their studies.

Previously, the association between hyperuricemia and osteoporosis was not fully understood pathologically, while some plausible explanations may be possible. At the physiological level, uric acid is considered an important endogenous antioxidant that scavenges reactive oxygen species (ROS) and mitigates cellular and vascular damage caused by oxidative stress^41^. Due to its antioxidant properties, uric acid may inhibit osteoclastic bone resorption and contribute to higher BMD^42^. In an in vitro study with mice, uric acid significantly suppressed osteoclastogenesis in a dose-dependent manner by reducing ROS production in osteoclast-precursor cells^17^. Additionally, a previous study found a significant positive correlation between serum 25(OH)D and serum uric acid levels^18^. However, uric acid also promotes oxidative stress by superoxide free radicals produced via NADPH oxidase (NOX4), and the imbalance between oxidative stress and antioxidation affects bone remodeling and causes osteoporosis^43^.

The present study has several limitations. First, we could not determine whether a causal relationship exists between hyperuricemia and osteoporosis due to its cross-sectional study design. Thus, further longitudinal studies adjusting for potential confounding factors are indicated to confirm our findings. Second, our data include a self-administered questionnaire and might have involved a recall bias of previous history. Third, we could not analyze the effects of physical exercise, waist circumference or the use of medication because this information was not available and could not be elicited due to the retrospective nature of our study. Furthermore, there was no questionnaire on gout in the survey. Additional longitudinal studies that include this information are needed. Fourth, according to the definition of osteoporosis, there may have been diagnosis errors because the diagnosis relied on the patient's memory. Diagnosis errors according to the definition of osteoporosis have already been pointed out in previous papers. In a paper comparing patients’ responses to diagnosis questions and T-scores, only 7.6% of men and 37.8% of women were aware of their diagnosis^44^. However, in studies with fragility fracture patients, the concordance rate between self-report and a clinical diagnosis of osteoporosis varied by country, ranging from 30 to 80%^45^. Among the countries, Korea showed highest rate of osteoporosis diagnosis. Osteoporosis had a prevalence of 6.8% in our paper, which is lower than that in previous reports (7.8 to 22.1%)^46,47^. Since our data showed a relatively high proportion of older men over the age of 60, we speculate that the overall number of participants with osteoporosis may have been low. Finally, the participants in this study included adults over 40 years old, so the findings might not be generalizable to younger people. Despite these limitations, the present study demonstrated that the associations between hyperuricemia and osteoporosis differ by age and sex group. We found a protective effect of hyperuricemia against osteoporosis, particularly in older women. While prior studies observed a positive association between serum uric acid and BMD, our study demonstrated the OR of hyperuricemia and osteoporosis. The examined participants were also a low-risk, asymptomatic large population of Koreans and had laboratory results, which is an additional strength of our study.

## Conclusion

In conclusion, our study showed that hyperuricemia was associated with a decreased risk of osteoporosis after adjusting for confounding factors. In subgroup analyses according to age and sex, statistical significance was observed only in the group of older women.

## Data Availability

Data in this study are from the Korean Genome and Epidemiology Study (KoGES; 4851-302), National Research Institute of Health, Centers for Disease Control and Prevention, Ministry for Health and Welfare, Republic of Korea. These data are available online with permission from the Division of Epidemiology and Health Index of the Korea Centers for Disease Control and Prevention.
